# Progress in Surgery

**Published:** 1897-12-11

**Authors:** 


### Progress in Surgery.

### SURGERY OF THE SPINE.

**Immediate Reduction of the Deformity in Pott's Disease.**—Towards the end of last year Calot[1] brought before the Académie de Médicine a new method by which he had been able to get rid of the deformity resulting after the cure of Pott's disease. His plan consists in forcibly undoing the deformity by making strong traction on the spine, assistants pulling in opposite directions, while the surgeon vigorously presses upon the *gibbosité*, until the displaced vertebræ have reached, or even gone beyond, the level of the neighbouring ones. In many cases, before applying this traction, he has excised the bony projection with the skin over it, and in a few has even removed a wedge-shaped piece of the posterior vertebral arch. When the spine is straight the patient is put up in a plaster of Paris jacket, extending from the head to the pelvis, and is retained in this for from four to six months, the appliance being removed and replaced several times during that period. For a further variable period the patient wears a less rigid form of "corset." Thirty-seven patients had been treated in this way at the time of reporting, without any accident. In one or two cases abscesses formed some months after the operation, and were treated successfully in the ordinary way.

Stimulated by the success of these cases, several other French surgeons have carried out this method of treatment. Brun,[2] in two recent cases, straightened spines under chloroform with immediate results which, to him, were satisfactory. The ultimate results, however, have still to be seen. Michaux[2] found the operation very easy in a nine-year-old boy, who had suffered from Pott's disease since he was three. In the evening of the operation, however, there developed a slight paresis of the left lower limb. Broca[2] has employed it in three cases with impunity, but does not feel sure of the general applicability and safety of the method. He knows of a case where peritoneal symptoms, presumably due to the rupture of an existing abscess, followed it. Jones and Tubby[4] found the operation easy in some cases in which they tried it. Menard[3] opposes the method as one fraught with danger, and his opinion is the result of operations made on the cadaver, and from a study of museum specimens. His main objection lies in the fact that the straightening of the bony column leaves a large gap, measuring from 2 to 6 centimetres, which, to effect a satisfactory cure, must get filled up with bony callus. Failing this, a soft fibrous mass is left, which weakens the spine, or, by contraction, reproduces the deformity. Monod is also against the treatment in old-standing cases for very much the same reasons. Noble Smith,[5] however, does not fear the failure of ossification in the gap left after straightening, on the strength of a case he had in which the bodies of several vertebræ were destroyed, and yet the arches kept up the integrity of the column. He suggests a danger from loosened fragments of bone acting as foreign bodies and causing irritation.

Brun, experimenting on the cadaver of a child who died while under treatment for Pott's disease, found the gap left after straightening to measure between 8 and 10 centimetres in vertical diameter. Like other observers, he found the cord and meninges intact. He favours the procedure in recent cases, but not in those of long standing.

Chipault,[6] after straightening the spine, supplements the fixation of it by silver wire sutures passed in figure-of-eight fashion between the healthy spinosus processes above and below the projection. In children he applies an orthopædic apparatus; in adults he places them on a hard mattress. Constitutional treatment is of the utmost value.

Noble Smith,[5] who refers to this method of treatment in an appendix to the second edition of his work on "Spinal Caries," says that although he has never, like Calot, attempted immediate and forcible reduction of these deformities, he has for years systematically done so by graduated pressure and traction of adaptable apparatus. His principle is entirely different, and consists in altering the curve above and below the disease. The "adaptable metal splint," by which he effects this, is fully described, and its method of application explained and illustrated in the fourth chapter of his book, and well deserves the attention of anyone interested in the treatment of cases of this kind.

**Laminectomy in Spinal Caries.**—In our last *résumé* of the recent literature of Pott's disease (The Hospital, June 5th, 1897) we referred at some length to this procedure. Noble Smith, in discussing the operation, points out some disadvantages involved in attempting to relieve paralysis by this means. He considers the bony arches of the vertebræ, which are seldom diseased, of great value in supporting the weight of the spine, and so relieving the inflamed and diseased bodies. He therefore deprecates the removal of these parts, on which so much depends. The interference with perfect rest which an operation entails, and the risk of injury to the membranes and cord, he also looks upon as serious objections. Nevertheless, he admits not only the necessity for laminectomy in some cases, but also its beneficial results. It is always imperative, however, that less heroic measures should have had a thorough trial.

Willard, of Philadelphia,[7] contributes a paper on the treatment of paraplegia in spinal caries by laminectomy. He has found in his experience of 30 years that treatment by rest and fixation seldom fails if thoroughly and patiently tried for at least a year or 18 months. While not condemning the operation, he considers the mortality so high and the prospect of permanent cure so uncertain as to limit its applicability to comparatively few cases. In 134 collected cases he found the immediate mortality to be 24 per cent., while 36 per cent. died within the first month. As regards results, about 65 per cent. either die or are not materially improved. It is to be borne in mind, however, that only the severest cases undergo operation, and in these few would recover without it. This surgeon considers the operation by no means an easy one. His general conclusions are: (1) Prognosis in pressure paraplegia is hopeful, unless the cord has been actually destroyed; (2) laminectomy should never be undertaken unless at least a year of persistent treatment by rest, fixation, and extension has been patiently tested; (3) the mortality and uncertainty of results are great; (4) the dangers of the operation are hæmorrhage, prolongation of the operation, and manipulation of the cord; (5) in spite of these risks, the operation has its place in surgery, and is justified in selected cases when persistent and carefully-applied measures have failed.

In discussing this paper, Abbe, Collins, and Starr agreed in the main with the writer of it.

[1] Franc. Méd., 1896, No. 52. [2] Med. Week, May 21, 1897. [3] Med. Week, 1897, p. 225. [4] Lancet, Jan., 1897. [5] Spinal Caries, 2d Ed., 1897. [6] Centralblatt Chir., Sept. 4, 1897. [7] Journal Ment. and Nerv. Dis., April, 1897

